# Artificial intelligence empowering public health education: prospects and challenges

**DOI:** 10.3389/fpubh.2024.1389026

**Published:** 2024-07-03

**Authors:** Jin Wang, Jianxiang Li

**Affiliations:** School of Public Health, Suzhou Medical College of Soochow University, Suzhou, China

**Keywords:** artificial intelligence, public health education, personalized education, interactive and immersive learning, public health crisis

## Abstract

Artificial Intelligence (AI) is revolutionizing public health education through its capacity for intricate analysis of large-scale health datasets and the tailored dissemination of health-related information and interventions. This article conducts a profound exploration into the integration of AI within public health, accentuating its scientific foundations, prospective progress, and practical application scenarios. It underscores the transformative potential of AI in crafting individualized educational programs, developing sophisticated behavioral models, and informing the creation of health policies. The manuscript strives to thoroughly evaluate the extant landscape of AI applications in public health, scrutinizing critical challenges such as the propensity for data bias and the imperative of safeguarding privacy. By dissecting these issues, the article contributes to the conversation on how AI can be harnessed responsibly and effectively, ensuring that its application in public health education is both ethically grounded and equitable. The paper’s significance is multifold: it aims to provide a blueprint for policy formulation, offer actionable insights for public health authorities, and catalyze the progression of health interventions toward increasingly sophisticated and precise approaches. Ultimately, this research anticipates fostering an environment where AI not only augments public health education but also does so with a steadfast commitment to the principles of justice and inclusivity, thereby elevating the standard and reach of health education initiatives globally.

## 1 Introduction

Public health education is the cornerstone for maintaining and improving community health standards, preventing diseases, and promoting healthy lifestyles. Public health education typically relies on five fundamental and core disciplinary areas, including biostatistics, epidemiology, environmental health sciences, social and behavioral sciences, and health policy and management (1, 2). Historically, traditional teaching methods have dominated the field of education, heavily dependent on didactic teaching methods, face-to-face interaction, and standardized curricula (3, 4). Although these methods are foundational, they have limitations in scalability, accommodating diverse learning needs, and rapid dissemination of information, particularly in the context of global health crises such as the COVID-19 pandemic in recent years (5).

Contemporary public health education faces several challenges that impact its effectiveness. A one-size-fits-all approach often fails to consider the diverse demographic and socio-economic backgrounds of learners, leading to gaps in health literacy (6). Moreover, the static nature of traditional educational resources can result in outdated information, restricting the responsiveness of public health education to emerging public health threats (1). At the same time, there is a growing demand to enhance the practical skills of public health professionals, and traditional lecture-based methods fall short of efficiently addressing this issue.

While facing these challenges, the emergence of Artificial Intelligence (AI) in educational reform heralds a new era of potential solutions. As an emerging technology of the 21st century, AI has penetrated various fields such as economy, science, and healthcare, becoming a significant driving force for future development (7, 8). Indeed, the World Health Organization emphasized in its 2018 report that digital technologies and AI are poised to become key tools in achieving its global strategic goals: to increase the number of people benefiting from universal health coverage by one billion, to increase the number of people protected from health emergencies by one billion, and to improve the health and well-being of one billion people (9). AI technologies can personalize learning experiences, process vast amounts of data to obtain the latest learning resources, and provide interactive training through simulations (10, 11). AI can effectively tailor educational content according to individual needs, thereby improving learners’ comprehension and retention rates (10). Furthermore, the application of AI in public health education is extensive, ranging from automating administrative tasks to facilitating the resolution of complex problems and decision-making processes in public health scenarios.

The prospects of AI promise to overcome the rigidity of traditional educational systems, offering opportunities for continuous lifelong learning and rapid adaptation to dynamic public health environments (12). By integrating AI into public health education, there is not only the potential to enhance the quality and effectiveness of education but also to innovate the tools and methods of imparting public health knowledge, ultimately equipping current and future public health professionals with the capabilities needed to meet the multifaceted health challenges of the 21st century. This study aims to elucidate the application of AI in public health education, with a focus on assessing the efficacy of personalized communication and interventions. The study will explore the scientific principles of AI, its future development, and especially its potential applications in education, behavioral modeling, and health policy formulation. Additionally, it will identify the challenges of data bias and privacy in AI applications and propose strategies to ensure ethics and equity, guiding intelligent and precise health interventions.

## 2 Theoretical foundations of AI in education

### 2.1 Key concepts and technologies

AI in education is a burgeoning field that employs advanced computational methods to support and enhance learning. AI refers to machines or software that can perform tasks requiring human intelligence, such as understanding natural language, recognizing patterns, and making decisions (13, 14). Machine Learning (ML), a subset of AI, enables predictions or decisions to be made by training on data, without being explicitly programmed for the tasks (15). This capability is particularly transformative in educational settings, as it allows the vast amounts of data generated to be leveraged for personalizing the learning experience to meet individual needs (9). Here we list some machine learning algorithms and their application scenarios involved in the field of medical education. These algorithms enhance learning efficiency and educational quality in medical education and practice, and provide new perspectives and tools for medical research, especially in optimizing disease diagnostics and treatment strategies (16–18).

**Table:** 

Algorithm name	Definition	Application scenario
Few-Shot Learning	A machine learning technique that enables a model to learn new tasks from a very limited number of examples.	Used in medical education for studying rare cases, such as rare disease diagnostics.
Transfer Learning	The process of using knowledge gained while solving one problem to solve a related but different problem.	Transferring knowledge between various medical imaging tasks, like from cardiology to oncology diagnostics.
Meta-Learning	Also known as “learning to learn,” it enables models to learn how to learn effectively across multiple tasks.	Developing intelligent diagnostic tools that adapt to various types of medical tests or treatment plans.
Reinforcement Learning	A method of learning that strives to maximize rewards through interactions with the environment.	Used for simulating clinical decision-making processes, like learning to choose the best treatment strategies in complex scenarios.
Self-supervised Learning	A training method where the model generates its own labels from the unlabeled data by predicting parts of it.	In medical imaging analysis, used for extracting useful features from unlabeled medical images, such as tumor detection.
Generative Adversarial Networks (GANs)	Comprising generator and discriminator networks that compete to generate increasingly realistic data.	Generating high-quality medical images for training, improving students’ accuracy in interpreting complex images.

Data analytics, another cornerstone technology, involves analyzing large datasets to discover hidden patterns, correlations, and insights (19). In the realm of education, data analytics can assist educators in understanding student learning processes and outcomes, enabling them to make more informed decisions about teaching strategies and interventions (20). The integration of these technologies is reshaping educational methods by facilitating adaptive learning platforms, intelligent tutoring systems that provide real-time feedback and support, and predictive analytics guiding educators in curriculum design and modification.

Pre-Training refers to the process of training a model on a large amount of general data before training it on a specific task. This step helps the model to establish a broad knowledge base that can be fine-tuned later to adapt to specific applications. In medical education, pre-trained models can be used to understand complex medical texts or assist in diagnoses, enhancing the efficiency and accuracy of subsequent tasks (21).

Prompt Engineering is an emerging field of research that involves designing and optimizing prompts (such as questions and instructions) input into AI models to improve model outputs. In medical education, effective prompt engineering can help educators create more effective AI teaching tools, such as simulated dialog systems, which can guide students more effectively through precise prompts (22).

### 2.2 Integration of learning theories with AI

The convergence of constructivist and cognitivist theories with AI represents the synergy between human cognitive development theories and computational algorithms (23). Constructivist learning theory posits that learners construct knowledge through experiences and reflection, while cognitivist theory focuses on the internal mental activities of the mind, considering understanding these processes as key to comprehending how learning occurs (23). AI can support constructivist approaches by providing simulated environments where learners can experiment and receive immediate feedback. Cognitivist strategies can be enhanced through AI’s capability to process and analyze data from learners’ interactions with content, thus revealing insights into cognitive processes. AI enables personalized learning pathways by analyzing data of individual learners and adjusting educational materials accordingly. This personalization ensures that each learner receives content that best suits their learning style, pace, and current understanding, optimizing the learning process (24).

One such case involves the use of AI to develop dynamic learning modules that automatically adjust content based on student interactions and progress. For example, if a student shows difficulty understanding a medical topic, the system provides additional resources such as video tutorials, interactive simulations, and customized practice questions to help overcome learning obstacles. Another case is the integration of AI-driven simulation technology in teaching to provide practical experience and immediate feedback. During the pandemic, this approach was particularly valuable as it allowed students to practice and refine their clinical skills in a safe virtual environment, thereby reducing physical contact and potential health risks.

### 2.3 Digital health

Digital health encompasses a broad range, including MedTech, wellness technologies, and clinical settings (25). In this manuscript, we focus on how these technologies are integrated into public health education, particularly under the prospects and challenges empowered by Artificial Intelligence.

MedTech typically includes tools and devices that use advanced technology to enhance diagnosis, treatment, and patient monitoring (26). These technologies play a crucial role in public health education, especially in teaching future healthcare professionals about new diagnostic tools and treatment technologies.Wellness technologies focus on tools that promote health and prevent disease, such as health trackers and personal health applications (27). In public health education, these technologies can be used to educate students on how to leverage these tools to improve health behaviors and outcomes for patients and the public. Clinical settings involve the application of digital health, including Electronic Health Records (EHR), Clinical Decision Support Systems (CDSS), and telemedicine services (28, 29). These technologies provide a practical platform for students to learn how to apply AI and other digital tools in real medical environments to enhance care efficiency and effectiveness.

The application of digital health in public health education demonstrates its wide coverage and potential. By integrating these technologies, we not only enhance the quality of education but also better prepare students to face modern healthcare challenges. Artificial Intelligence, as a core technology in this process, enhances the effectiveness of these applications, driving innovation and development in public health education.

## 3 Health education challenges

### 3.1 Challenges in medical education

Prior to and following the COVID-19 pandemic, significant changes occurred in the education of healthcare professionals. A decade of innovation, exemplified by massive open online courses, was disrupted by the COVID-19 pandemic, which unsettled educational systems worldwide. This increased the utilization of online technologies, led to substantial institutional reorganizations to accommodate a blended model of online and face-to-face teaching, and exposed pre-existing inequalities in access to educational resources both within and between nations (30). In addition to sparking an economic and social crisis, the pandemic caused numerous disruptions that could have long-term effects on both education and healthcare systems. Furthermore, global medical education and healthcare professions are facing an array of additional challenges (30–33) (Table 1).

**Table 1:** Challenges facing global medical education and health professions.

Challenges in education and training	Challenges in clinical practice and public health	Challenges in technology and innovation
Resource Scarcity	Aging Populations	Digital Health and Telemedicine
Technological Adaptability	Chronic Disease Management	Multicultural Healthcare Needs
Educational Quality Disparity	Global Health Inequities	Cultural Differences
Continuing Medical Education	Disease Prevention and Health Promotion	Ethical and Legal Issues
Interdisciplinary Integration	Pandemic Response	
Environmental Health Challenges	Healthcare Workforce Shortage	
Medical Ethics Education	Patient Safety and Healthcare Quality	
Research and Innovation Capacity	Public Health Strategies and Policy Making	

### 3.2 How AI addresses these challenges

AI plays a pivotal role in tackling global healthcare challenges. The World Health Organization (WHO) recognizes health technologies and AI-based products as integral to healthcare services, aiming to ensure that an additional one billion patients have access to insurance, emergency care, and enhanced health status (9, 34). AI assists healthcare providers by facilitating disease diagnosis, clinical reasoning, data analysis, and informed decision-making (35). Beyond traditional uses in imaging and pathology, AI applications now span diverse medical specialties such as rheumatology, neurology, endocrinology, orthopedics and surgery areas which are at the forefront of medical research advancements.

## 4 Artificial intelligence in education and public health

### 4.1 Adaptive learning

Adaptive learning is a highly personalized educational approach designed to adjust teaching content and methodologies based on the learner’s needs, abilities, and progress. In the context of human learning, this means that the educational systems are capable of recognizing a student’s learning preferences, knowledge level, and obstacles to learning, thereby offering a customized learning experience (36). For example, adjusting the level of difficulty, learning materials, and forms of assessment based on the student’s responses and interactions to ensure maximum efficiency and benefit along their personal learning journey.

### 4.2 Artificial intelligence in education

**Figure 1:**
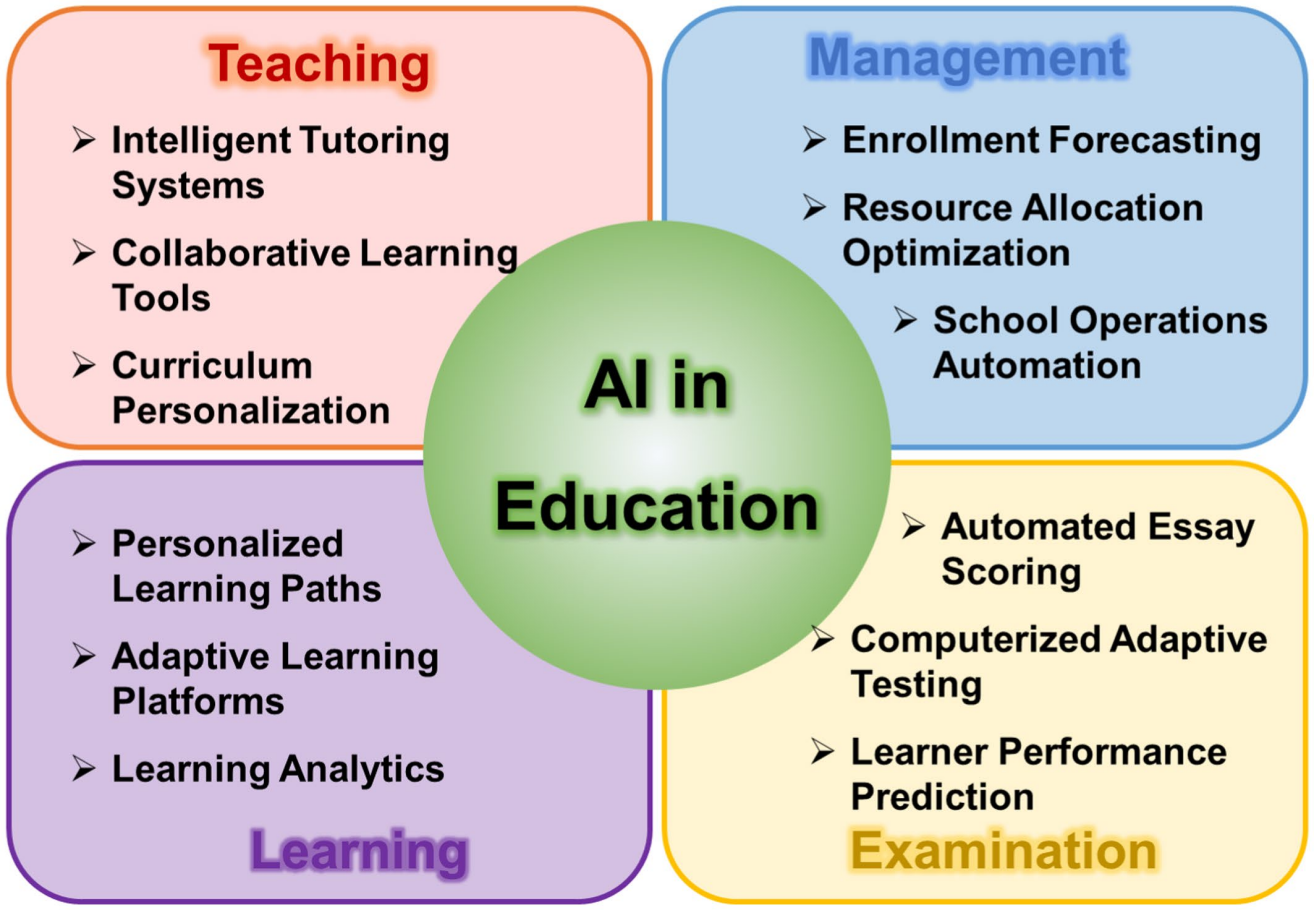
Application scenarios of AI in education according to the summary of the literature.

The incorporation of AI in education has transformed teaching and learning. Simulation-based immersive experiences are now central in educational settings, providing significant advancements in student engagement (11). AI provides new opportunities, potential, and challenges for innovation in education, encompassing innovations and developments in teaching, management, learning, and assessment across these four facets (Figure 1) (37, 38). AI’s capacity to customize learning materials is unprecedented, enabling a previously unattainable level of personalization. It monitors student interaction and delivers targeted feedback where it is most impactful. However, this progress is not without concerns. The ease with which students can generate assignments using AI prompts critical questions about the essence of learning and the assessment of genuine understanding, ensuring it reflects the student’s knowledge rather than a byproduct of technology (11). Feng and Law conducted a systematic review of literature on artificial intelligence in the field of education from 2010 to 2019, revealing popular keywords in the field, including neural networks, deep learning, eye tracking, and personalized learning (39).

### 4.3 Artificial intelligence in public health

One of the primary tasks in optimizing public health systems is the integration of information technology and data science to strengthen disease prevention and health monitoring capabilities. This monitoring involves systematic and continuous collection, management, analysis, and interpretation of data, aimed at stimulating and guiding specific preventive measures. The application of big data analytics and AI can facilitate the early detection of epidemics and other public health risks, providing support for the implementation of targeted and precise public health strategies (40–42). AI’s capability to sift through and make sense of extensive health data can revolutionize public health and disease tracking, setting the stage for more targeted health strategies. The examination of vast amounts of health information from diverse sources about individuals, locations, and times could offer deeper and broader perspectives on what causes illnesses, tailoring insights to both individual care and community health. This could speed up disease monitoring and inform the development of health policies and their execution (43). Incorporating cultural considerations into the AI frameworks used in public health is imperative for ensuring that these technologies respect individual preferences and promote equity. As highlighted by the OECD, responsible AI in health must be adaptable to change, respect individuals, champion equity, and achieve better health outcomes for all (44).

**Figure 2:**
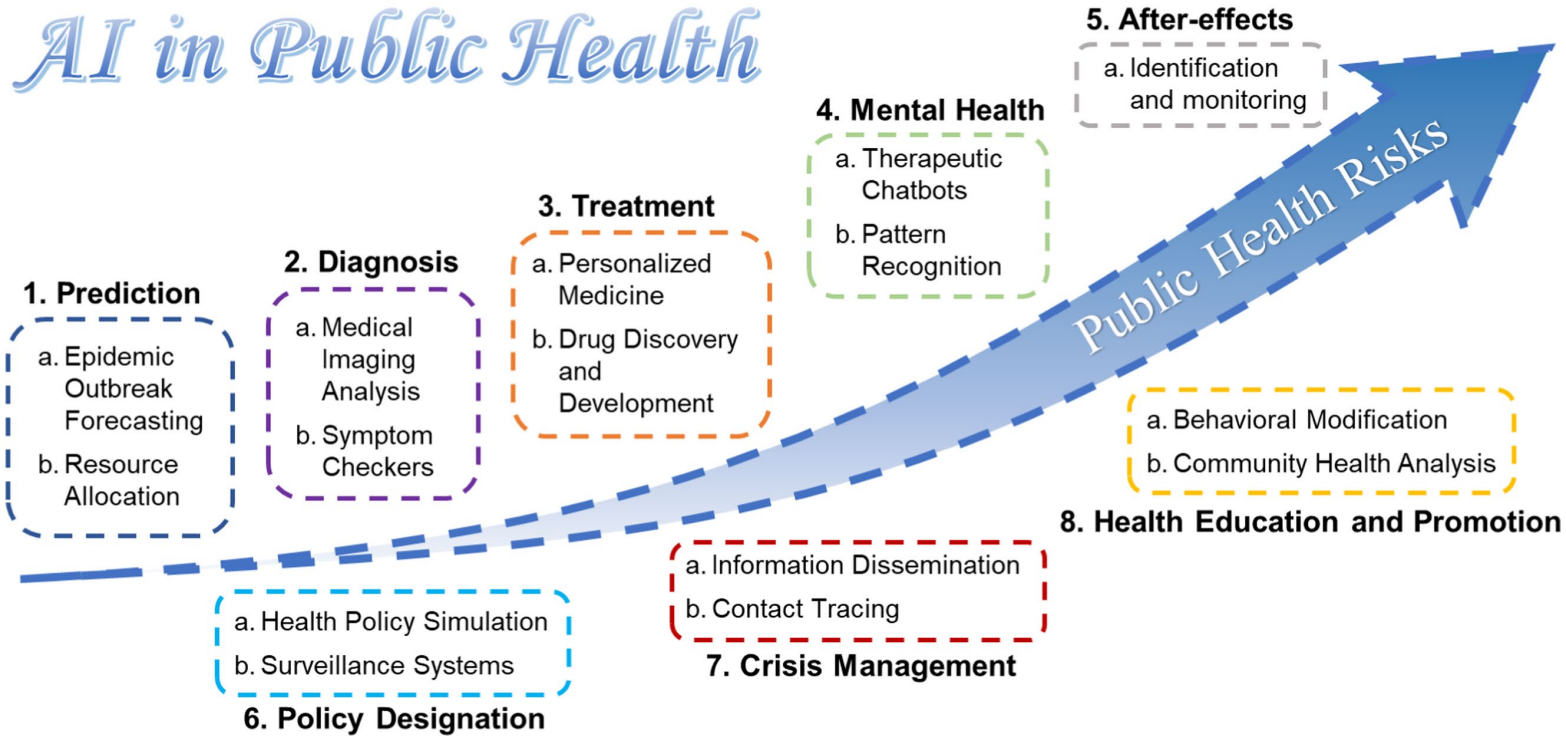
Application scenarios of AI in public health risks according to the summary of literature.

Based on the literature, we have summarized the following 10 application scenarios of AI in the field of public health (Figure 2) (42, 45, 46). For example, during peacetime preceding public health crises, it is imperative to harness AI models to integrate public health data from various regions to enhance forecasting capabilities, thereby achieving rapid response. Additionally, AI should be leveraged for timely resource allocation based on predictive outcomes. Furthermore, AI can facilitate the swift diagnosis and personalized treatment of patients during crises, as well as support post-crisis mental health education and the monitoring of related sequelae (46). For management and decision-making bodies, AI can rapidly provide the most sensible policy recommendations based on model data (45).

### 4.4 Education vs. practice in public health

In the field of public health, there is a clear distinction between the educational and practical applications of Artificial Intelligence (AI). On the educational level, AI is primarily used to enhance learning experiences and improve educational outcomes, such as training medical students and professionals through simulations and adaptive learning systems. On the practical level, AI applications focus more on the analysis of public health data, disease prediction models, and the formulation and execution of health strategies. For example, AI can help predict disease outbreaks by analyzing historical health data, or improve the efficiency of responses to public health crises by optimizing resource allocation.

### 4.5 Current implementation of AI in public health education

Simulation exercises are recognized as an indispensable part of preparedness in addressing emergency public health crises by health departments (47). The use of simulations to create immersive scenarios that reflect real-world crises has gained widespread recognition within disaster management. Such simulations enhance organizational capabilities to respond to major emergencies, particularly health crises related to infectious diseases (48, 49). Historical emergency events have highlighted the need for even the most experienced professionals to engage in continual learning to respond safely and effectively to emerging and re-emerging health challenges. Strengthening our preparedness strategies is vital for ensuring swift and effective emergency responses (50, 51). In recent years, several AI-driven initiatives have been introduced in the field of public health education. AI-driven platforms are used to simulate public health crises, allowing students to engage in problem-solving exercises that emulate real-world scenarios. These platforms utilize machine learning algorithms to adjust scenarios based on learners’ decisions, providing a dynamic learning experience. A recent study reviewed the barriers and potential strategies to simulation and technology-enhanced learning during the COVID-19 crisis in China. They identified that once educational needs were pinpointed, simulations emerged as a powerful tool against the virus. This approach played a key role in providing practical training for frontline medical workers, ensuring patient safety, and offering a safe environment for healthcare professionals to gain practical experience in managing COVID-19 (52). The authors advocate for the creation of dedicated simulation-based courses to better equip medical workers for current and anticipated pandemics (52).

## 5 The future of AI in public health education

As we delve into the intersection of public health and technology, AI emerges as a transformative force within public health education. It offers an unprecedented opportunity to enhance the field through its impact on future curricula and teaching methodologies. This section explores the integration of predictive analytics and personalized learning into public health education, as well as the rapid development of interactive and immersive learning experiences facilitated by AI.

### 5.1 AI-driven curriculum development and knowledge dissemination

Incorporating AI into the development and delivery of public health curricula represents a revolutionary step, providing a dynamic and responsive educational model (53). The analytical prowess of AI enables the identification of emerging public health threats and trends, ensuring that educational content remains timely and relevant. Concurrently, AI systems excel at aligning this content with established educational standards, thus preserving academic integrity and rigor. Beyond content creation, AI enhances personalized learning experiences by adapting to individual students’ pacing and comprehension levels, optimizing the educational process. Such adaptive learning technologies adjust content difficulty and teaching styles based on the learner’s proficiency, fostering an efficient and effective learning process (54).

AI’s influence extends to learner engagement, using interactive technologies like conversational agents and immersive simulations to deepen student involvement. These AI-supported interactive platforms have proven effective in enriching the learning experience and enhancing student engagement (55). Furthermore, AI’s adaptability in presenting educational materials in various formats, such as visual, auditory, and kinesthetic, caters to diverse learning preferences, significantly increasing knowledge retention (56). This rich AI educational framework not only fosters a more engaging and personalized learning environment but also ensures that the workforce entering the public health sector is equipped with up-to-date knowledge, ready to apply it effectively in a rapidly evolving context.

### 5.2 Integrating AI into public health education

To effectively integrate AI technology into public health education, we propose a blended curriculum that includes both theoretical courses and practical training using virtual simulations of real-world data. The theoretical component would cover the basics of data science, public health theory and applications of AI in public health, such as disease pattern recognition and health data analysis. The practical component would focus on allowing students to work with real-world public health data in a controlled environment through advanced simulation platforms, such as simulating epidemic outbreaks and optimizing vaccine distribution strategies.

Methods for assessing learning outcomes should include a combination of qualitative and quantitative evaluations. Quantitative assessments could measure students’ mastery of theoretical knowledge through traditional exams and tests, while qualitative assessments could examine practical application skills through project-based evaluations. Additionally, peer reviews and self-assessments should be included to gain a comprehensive understanding of students’ innovation abilities and critical thinking levels when solving real public health issues. Such an assessment system would not only track students’ learning progress but also enhance their ability to apply AI technologies in real-world settings.

### 5.3 Measuring the efficiency and effectiveness of learning processes

**Figure 3:**
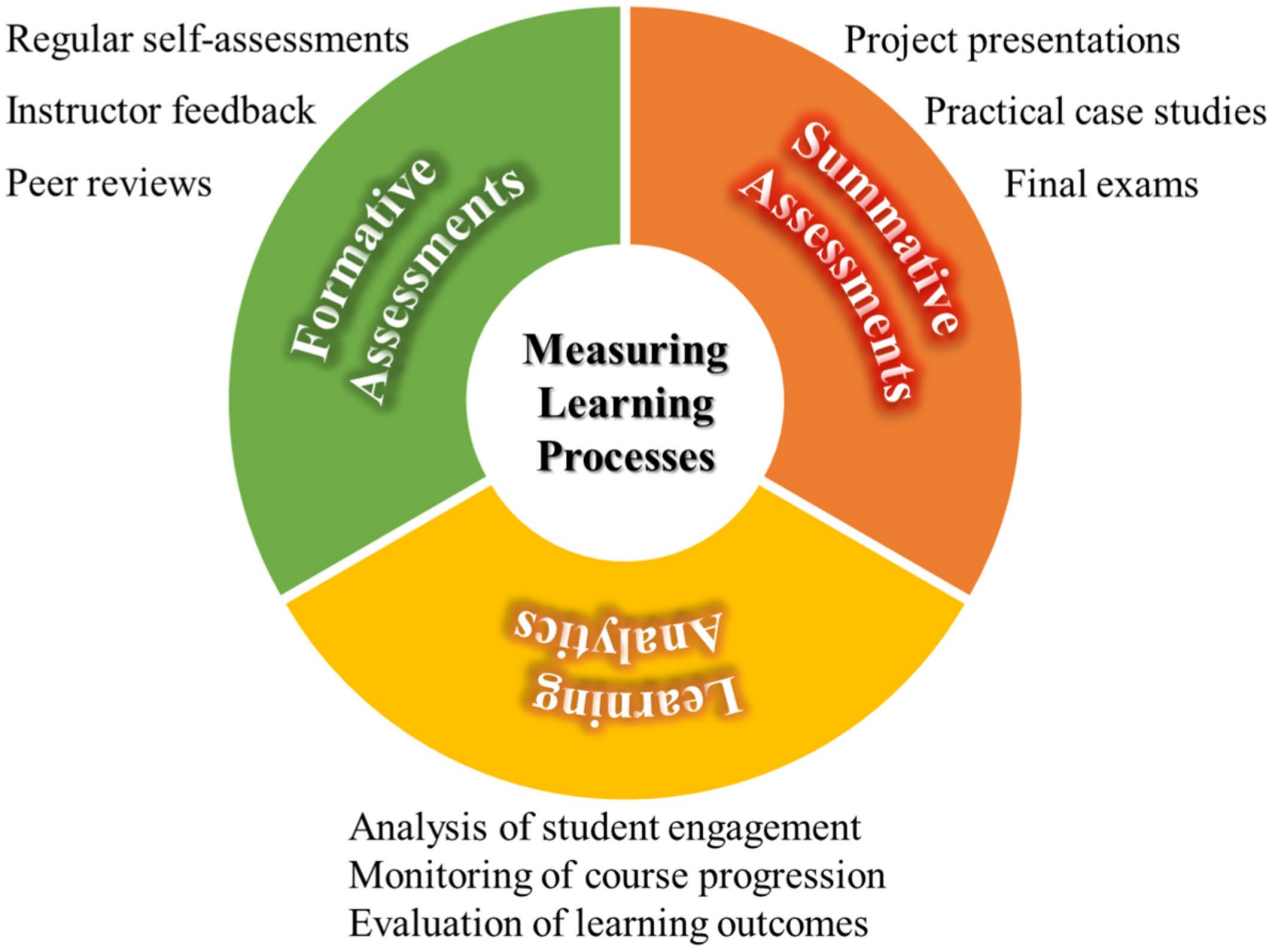
Measuring the efficiency and effectiveness of learning processes.

To effectively measure the efficiency and effectiveness of learning processes, it is recommended to employ a variety of assessment tools and metrics to ensure that educational activities meet the intended educational objectives (Figure 3). Firstly, formative assessments should be used to monitor the learning process and provide immediate feedback, allowing students to adjust their learning strategies in real time. Formative assessments not only evaluate students but also motivate them through feedback, enhancing their learning experiences (57). In medical education, formative assessments have been shown to improve scores in summative assessments of pathophysiology (58). However, to fully realize the potential of these techniques, integrating Artificial Intelligence (AI) is essential. This integration is subtly transforming medical education patterns, allowing students to engage in realistic medical scenarios without the constraints of the physical world.

Secondly, summative assessments are recommended to evaluate students’ learning outcomes at the end of the course. This can be achieved through final exams, project presentations, or practical case studies. Through these methods, educators can assess whether students have achieved the learning objectives of the course and have mastered the necessary knowledge and skills.

Additionally, it is advised to use learning analytics tools to collect and analyze data about learning activities, which can help educators understand overall trends and patterns in learning activities. Learning analytics can provide insights into student engagement, course progression, and learning outcomes, thereby enabling educators to make data-driven decisions to optimize teaching methods and curriculum design (59).

By combining these strategies, a comprehensive system for assessing learning efficiency and effectiveness can be established, providing a clear learning roadmap for students and educators to achieve higher educational success.

### 5.4 Predictive analytics and customized learning

Emerging AI technologies in the field of public health education are primarily manifested in predictive analytics capabilities, which can anticipate health trends and educational needs. Predictive analytics, powered by machine learning techniques, analyze historical and current data to make identifying potential future outcomes and trends possible. This capability is invaluable in the field of public health. It can predict epidemiological patterns, medical service demands, and emerging public health threats (60). For example, during a pandemic, Google AI developed a model that could predict the number of COVID-19 cases in a region 2 weeks in advance (61, 62). The impact on educational needs is profound; by understanding the challenges that may arise in the future, curricula can be designed proactively to equip students with the necessary knowledge and skills. This forward-looking approach ensures that public health professionals are prepared to address contemporary health issues at any time. Developing AI systems for these anticipated trends requires a complex fusion of data science and pedagogical expertise. AI algorithms must be trained on a broad range of data, from global disease outbreaks to local health service utilization patterns, to create content that is both relevant and timely (63). Customized learning content can then be dynamically adjusted to reflect the current state of public health affairs, ensuring that educational materials are not only contemporary but also predictive of future demands.

### 5.5 Interactive and immersive learning experiences

“Augmented Reality” (AR) refers to the process of creating a synthetic environment that ideally integrates the digital and physical worlds, applicable to applications for smartphones and computers, which use augmented reality to overlay computer-generated images onto the physical environment. “Virtual Reality” (VR) refers to any technology-created external environment simulation, combining computers and sensory devices such as headphones and gloves, which can create simulations very close to the real world, effectively immersing the audience “within it” (64). VR is not just for entertainment and gaming; it has broad applications in education, science, and training. To fully harness the potential of these technologies, it is critical to integrate the capabilities of Artificial Intelligence (AI), a process that is quietly transforming medical education paradigms (65), allowing students to engage in realistic medical scenarios without the constraints of the physical world.

In the short term, integrating AI into VR and AR simulations can immediately enhance the adaptability and interactivity of educational tools, allowing students to benefit from a more personalized and responsive learning experience. In the long term, this integration has the potential to radically transform the paradigm of public health education by providing highly customized and dynamically adaptive learning environments, making education more equitable and accessible.

**Figure 4:**
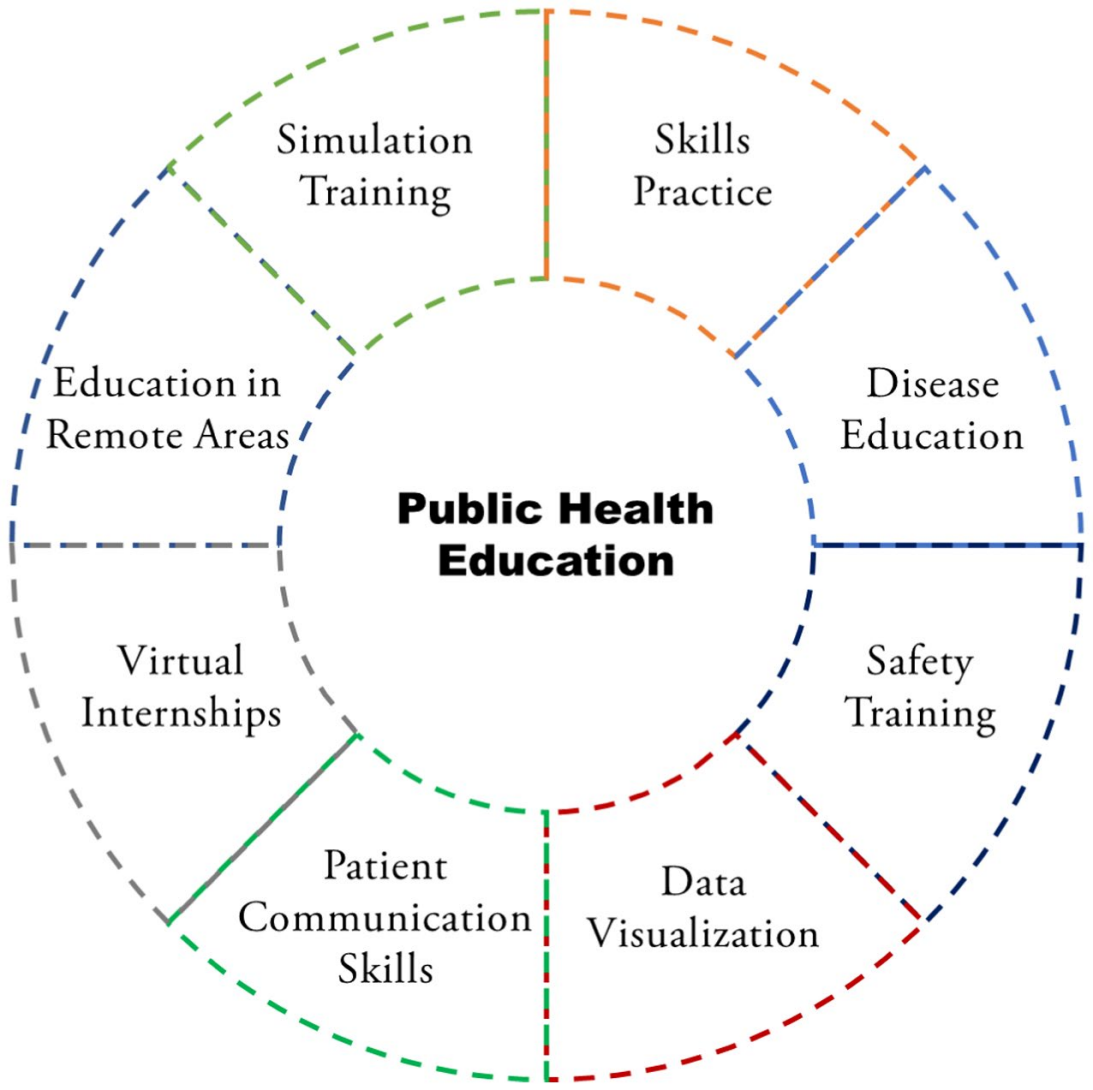
Application scenarios of AI in public health risks according to the summary of the literature.

In summary, the fusion of AR and VR with AI technologies holds broad prospects for application in public health education, including enhancing learning experiences, providing opportunities for practical training, strengthening behavioral change interventions, and contributing to educational equity (Figure 4) (66–69). As these technologies continue to evolve and improve, they are expected to promote more efficient and equitable public health education practices globally.

### 5.6 Applications of AI in public health management

In order to provide clearer insights into the short-term and long-term potential of Artificial Intelligence (AI) for public health authorities, it is essential to delve into specific use cases of AI in public health education. In the short term, AI technologies can significantly enhance the quality of medical education through simulation training courses and personalized learning experiences. For instance, AI-based simulation platforms can offer virtual reality surgical training, allowing students to practice and master complex medical procedures in a fully simulated environment, thereby improving their clinical skills without any risk. In the long term, the integration of AI will make the formulation and execution of public health policies more precise and efficient. AI can analyze vast amounts of health data to identify disease transmission trends, optimize resource allocation, and support public health strategies with data, thereby preventing outbreaks and enhancing the overall responsiveness of health systems.

## 6 Challenges of AI application in public health education

Recognized risks of AI include biases, privacy and security issues, discrimination, lack of transparency, inadequate oversight, job displacement, impersonalization, and the misapplication of context-specific algorithms (44). These risks are particularly pronounced in the field of education, especially in public health education.

### 6.1 Considerations of data privacy, ethics, and security

As we delve deeper into the application of AI in education, it becomes crucial to rigorously assess issues of data privacy, ethics, and security. Deploying AI systems in medical education involves handling sensitive data, necessitating stringent privacy protocols to prevent data breaches (70). Ensuring explicit consent from learners and securing their data with robust encryption should be fundamental principles within the AI educational framework. Moreover, AI systems must be designed to allow learners to opt-out easily and to understand transparently how their data is being managed and utilized. In light of these considerations, UNESCO has published “Guidance for the Use of Generative AI in Education and Research,” aiming to support immediate action by nations, plan long-term policies, and develop human capacities to ensure that these new technologies are human-centric (71).

### 6.2 Bias, fairness and cultural concerns

In the field of public health, AI applications offer unprecedented possibilities for large-scale public health initiatives and personalized health education, yet they also bring challenges associated with biases. AI models may perpetuate or amplify existing biases due to reliance on datasets that may contain historical biases or unrepresentative samples during algorithmic training, leading to unfair educational content and health advice for certain groups (72). For instance, Obermeyer et al. recently identified significant racial bias against Black patients in a widely used AI algorithm (73).

Given the significant influence of cultural backgrounds on educational programs and learning approaches, recognizing the diverse impacts of AI across different regions is crucial. We propose that AI technology can better serve a globally diverse population by adopting cross-cultural sensitive approaches in algorithm training and data collection. This includes developing culturally adaptable health education materials and ensuring that health data reflects a wide range of demographic characteristics and health needs. Such approaches not only promote the globalization and personalization of public health education but also ensure fairness and inclusivity in its application (74).

In light of these inherent challenges in AI, educators are required to explicitly teach about potential biases and their possible impacts on decision-making, critically assess outputs, and use technical and methodological means to identify and mitigate these biases. Through simulation games, students experience firsthand how AI operates, including its limitations and biases, thus gaining a better understanding of how to detect and correct biases in real-world applications. These strategies are designed to develop students’ critical thinking skills, enabling them to effectively identify and address issues encountered when using AI, thereby enhancing their professional capabilities in fields such as public health.

### 6.3 Necessary policy frameworks

To achieve these ethical standards, robust policy frameworks are essential. These frameworks should offer guiding principles for ethically sound AI use in educational settings, balancing the need for innovation potential with the protection of learners’ rights. They must also foster interoperability among different educational platforms, allowing harmonious integration of AI tools to serve a diverse learner population. “Artificial Intelligence and Education: Policy Guidance for Policymakers” published in 2021, addresses key policy issues, and lessons from analysis, and shares principles for people-centric policies, aiming to assist governments and partners in deploying AI effectively to transform educational and training systems for the collective good and a sustainable and inclusive future (75).

### 6.4 Collaboration between educational institutions, policymakers, and AI developers

Furthermore, collaboration among educational institutions, policymakers, and AI developers plays a crucial role in shaping the future of AI in public health education (76). Educational institutions provide valuable perspectives on teaching needs and student engagement, while policymakers play a key role in developing regulations for AI applications that align with societal norms and legal requirements. On the other hand, AI developers contribute the technical expertise necessary to create and refine AI tools for educational purposes.

These stakeholders must collectively participate in ongoing dialog to ensure responsible and effective implementation of AI in public health education. By fostering a cooperative environment, the educational sector can leverage AI to create innovative personalized learning experiences while upholding the highest ethical standards. The joint effort to align AI integration with ethical considerations and policy impacts is not only a regulatory requirement but also a moral responsibility to advance public health education to improve societal well-being.

## 7 Conclusion

In sum, the hallmark of future public health education will be a symbiotic relationship between AI and education. Predictive analytics will assist in forecasting and addressing the needs of public health workers, while AI-driven content customization will ensure that educational materials remain at the forefront of current and future public health challenges. The emergence of interactive and immersive learning experiences through AR/VR and AI will further solidify technology’s role in shaping the skills and competencies of future public health professionals. The integration of these technologies not only enhances public health training but also ensures that our responses to public health threats are informed, agile, and effective.

Looking ahead, the potential of AI in public health education is boundless, promising to deliver more responsive, engaging, and impactful education in line with the evolving global health landscape. Educators, technologists, and policymakers bear the responsibility of thoughtfully guiding this integration, ensuring that the deployment of AI in education enhances rather than diminishes the human elements in learning and public health practice.

## Data availability statement

The original contributions presented in the study are included in the article/supplementary material, further inquiries can be directed to the corresponding author.

## Author contributions

JW: Conceptualization, Writing – original draft, Writing – review & editing, Data curation, Methodology, Resources, Software, Visualization. JL: Conceptualization, Writing – original draft, Writing – review & editing, Funding acquisition, Investigation, Project administration, Supervision, Validation.
